# A Mechanistic Model of the HIF-1/HIF-2 Switch Regulating Hypoxia-Induced Cancer Stemness

**DOI:** 10.3390/ijms27114697

**Published:** 2026-05-23

**Authors:** Haiyue Zhan, Ping Wang, Feng Liu

**Affiliations:** 1 National Laboratory of Solid State Microstructures, Department of Physics, Collaborative Innovation Center of Advanced Microstructures, Institute for Brain Sciences, Nanjing University, Nanjing 210093, China; 2 Key Laboratory of High Performance Scientific Computation, School of Science, Xihua University, Chengdu 610039, China

**Keywords:** long-term hypoxia, HIF-1/HIF-2 switch, tumor cell stemness, OCT4 regulation, mathematical modeling, dynamics

## Abstract

A common hypoxic scenario in tumors involves unresolved acute hypoxia that eventually leads to sustained (chronic) hypoxia. This shift drives a characteristic “HIF switch”, where the key hypoxia-responsive factors change from HIF-1α to HIF-2α over time, and importantly, this switch is closely linked to stemness regulation. However, the mechanisms underlying this switch and its impact on stemness regulation are not yet fully understood. Here, we developed a mechanistic network model integrating the HIF-1/HIF-2 signaling axis with the stemness regulators OCT4 and SOX2. We found that the duration and intensity of hypoxia jointly shape the dynamics of HIF-1α and HIF-2α, ultimately regulating OCT4-mediated stemness. Under physioxia, HIF-2α–mTORC2 positive feedback supports the gradual accumulation of HIF-2α toward a modest steady level and low OCT4 expression, corresponding to a primed state. Under prolonged mild hypoxia, the concurrent induction of HIF-1α, albeit at low levels, and accelerated accumulation of HIF-2α elevate OCT4 to intermediate levels, promoting stem-like traits. Under moderate hypoxia, PHD-2-mediated negative feedback triggers pulsatile HIF-1α dynamics, driving a shift toward HIF-2α dominance. Ultimately, cooperative HIF-1α/HIF-2α signaling induces REDD1 and suppresses mTORC1-dependent protein synthesis, pushing OCT4 into a high-expression state associated with differentiation. This work presents a unified framework for understanding how the HIF signaling hierarchy coordinates metabolic and transcriptional programs to direct cell fate across varying hypoxic landscapes.

## 1. Introduction

Hypoxia is a common feature in solid tumors and exerts profound effects on tumor initiation, progression, and metastasis [1]. Owing to aberrant vascular architecture, unstable perfusion, and an imbalance between oxygen supply and demand driven by rapid tumor cell proliferation, the oxygen partial pressure within tumor tissues exhibits marked spatiotemporal heterogeneity, resulting in multiple hypoxic patterns, including acute, chronic, and cycling hypoxia [2]. Under specific conditions, inadequately resolved episodes of acute hypoxia, arising from fluctuations in blood perfusion or transient vascular occlusion, can promote the establishment of sustained hypoxia, thereby driving a temporal transition toward chronic hypoxia [3,4,5]. Importantly, this phased pattern of hypoxia, from acute to chronic, has been found to affect multiple processes, including embryonic vascular and skeletal development [6], and especially, orchestrate the acquisition of adaptive, stem-like phenotypes in tumor cells [7,8].

Tumor cells adapt to hypoxic microenvironments primarily through hypoxia-inducible factors (HIFs). HIFs, including HIF-1, HIF-2, and HIF-3, are heterodimeric transcription factors composed of an oxygen-labile HIF-α subunit (HIF-1α, HIF-2α, or HIF-3α) and a constitutively expressed HIF-1β subunit [9]. Under normoxia, prolyl hydroxylase domain proteins (PHDs) hydroxylate conserved proline residues within HIF-α, enabling recognition by the von Hippel–Lindau (VHL) protein and subsequent ubiquitination by the E3 ubiquitin ligase complex, ultimately targeting HIF-α for proteasomal degradation [10,11,12]. Under hypoxia, this hydroxylation is inhibited, allowing HIF-α to accumulate and translocate into the nucleus, where it dimerizes with HIF-1β to form a transcriptionally active complex that recruits co-activators and activates hypoxia-responsive gene expression [13]. While the function of HIF-3α remains incompletely understood, the functional divergence between HIF-1α and HIF-2α has attracted considerable attention. During the temporal progression of hypoxia from acute to chronic states, these two isoforms display distinct yet coordinated roles. Under acute hypoxia, HIF-1α rapidly stabilizes to drive metabolic reprogramming, primarily by enhancing glycolytic flux [8,14,15,16]. By contrast, HIF-2α becomes more stable and dominant during prolonged moderate hypoxia, shifting the cellular program toward proliferation, angiogenesis, and metastasis [3,6,17,18]. This temporal transition from an HIF-1α-dominated acute hypoxic response to a HIF-2α-driven long-term adaptive program is commonly referred to as “HIF switch” [6]. Elucidating the molecular basis of this HIF-1-to-HIF-2 transition is essential for understanding the shift from a survival-oriented stress response to malignant progression.

Considerable progress has been made in elucidating the molecular drivers of the HIF switch. Evidence suggests HIF-1-dependent metabolic reprogramming partially reactivates PHDs and, together with differential HIF1A and EPAS1 mRNA stability, drives a rapid decline in HIF-1α, whereas HIF-2α gradually predominates due to its greater stability, sustained synthesis, and reduced sensitivity to PHD-dependent degradation, thereby driving the HIF switch [19]. Consistently, in several cellular models the transcription and mRNA levels of EPAS1/HIF-2α increase progressively during hypoxia. This phenomenon has been associated with enhanced histone H3/H4 acetylation in the proximal promoter of HIF-2α, which promotes sustained HIF-2α transcription under prolonged hypoxia, while HIF-1α transcription becomes suppressed to varying degrees [3,19,20]. In addition, HIF-2α can induce the expression of epidermal growth factor (EGF), which in turn activates the Phosphoinositide 3-kinase (PI3K) signaling pathway, thereby enhancing the synthesis of HIF-2α [21,22]. Despite these insights into protein stability, transcriptional control, and signaling feedback, a unified quantitative framework that integrates these multi-layered processes to elucidate the systems-level logic of the HIF switch remains lacking.

Increasing evidence indicates that the hypoxic microenvironment promotes cancer stemness by reprogramming transcriptional and regulatory networks, with metabolic shifts functionally integrated into this process [18,23,24]. Hypoxia can reactivate the OCT4 promoter, enabling differentiated cells to revert to a stem-like state. HIF-2α can promote stemness-associated transcriptional programs by directly regulating OCT4 and, in specific contexts, by modulating NANOG and SOX2 expression [25,26]. The stemness-associated metabolic reprogramming is primarily mediated by HIF-1-regulated glycolysis [15,16,18]. Specifically, HIF-1-driven glycolysis elevates intracellular NADH levels, which activate C-terminal binding proteins (CtBPs) that function as transcriptional coactivators to enhance the expression of stemness factors such as OCT4, thereby coupling HIF-1 signaling, glycolysis, and stemness regulation [14,27,28]. This coupling is further strengthened by a reciprocal link where GLUT1, a canonical HIF-1 target, is also transcriptionally regulated by OCT4 [29]. Thus, while both HIF-1 and HIF-2 cooperatively regulate stemness through distinct metabolic and transcriptional mechanisms, the systemic logic underlying this coordination remains to be elucidated [8,14].

Theoretical models have been developed to describe how hypoxic signaling regulates the dynamics of HIF-1α and to elucidate the molecular mechanisms through which HIF-1 drives diverse hypoxic responses [30,31,32,33,34,35,36]. By contrast, quantitative models describing HIF-2 regulation remain relatively limited. In particular, mechanistic dynamical models addressing the HIF switch and quantitative frameworks linking this transition to the acquisition of cellular stemness are still lacking.

To address these questions, we developed a regulatory network integrating the HIF-1/HIF-2 signaling axis with the stemness regulators OCT4 and SOX2 to investigate the molecular dynamics underlying the HIF switch and its role in tumor cell stemness. We show that the shift from HIF-1α toward HIF-2α dominance under non-severe hypoxia arises from the interplay of two distinct mechanisms: slow HIF-2α accumulation driven by a positive feedback loop, and pulsatile HIF-1α dynamics induced by a negative feedback loop. We identify hypoxia severity as a critical determinant of a triphasic OCT4 state transition, elucidating how distinct feedback loops drive cells from a primed state under physioxia, to a stem-like state under mild hypoxia, and eventually toward differentiation under moderate hypoxia. These results highlight the profound impact of HIF signaling on governing cancer cell plasticity.

## 2. Results

### 2.1. Long-Term Hypoxia-Induced Transition from HIF-1 to HIF-2

**Figure 1:**
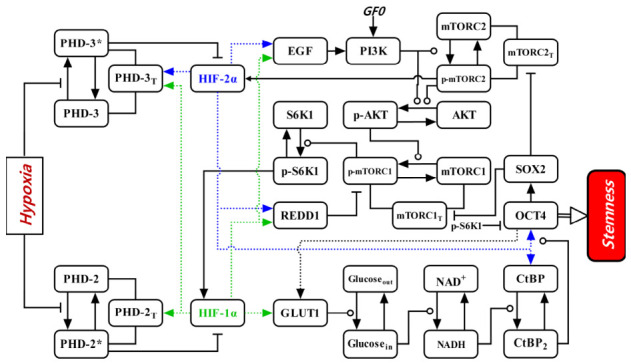
Schematic of the integrated regulatory network. Hypoxia is sensed through PHD-2- and PHD-3-mediated degradation of HIF-1α and HIF-2α, respectively. Growth factor (GF_0_) signaling modulates the synthesis of both HIF isoforms via the PI3K–mTORC1/2–S6K1 axis. Notably, HIF-2α is embedded within a HIF-2α–EGF–PI3K–mTORC2 positive feedback loop. Downstream, the stemness regulator OCT4 is co-regulated by the HIF-1/2 axis, protein synthesis signaling (mTORC1), and glycolytic metabolism (GLUT1 and CtBP_2_). Asterisk (*) indicates the active form. HIF-mediated transcriptional regulation is denoted by dashed lines. State transitions are indicated by solid lines with arrowheads. Activation and inhibition are represented by round- and bar-headed lines, respectively, while other processes are denoted by hollow arrows.

An integrated network model including HIF-1α, HIF-2α, and their downstream stemness regulators OCT4 and SOX2 was developed to investigate the mechanisms underlying the HIF switch and its regulation of tumor cell stemness (Figure 1). The modeling details are presented in Section 4.

**Figure 2:**
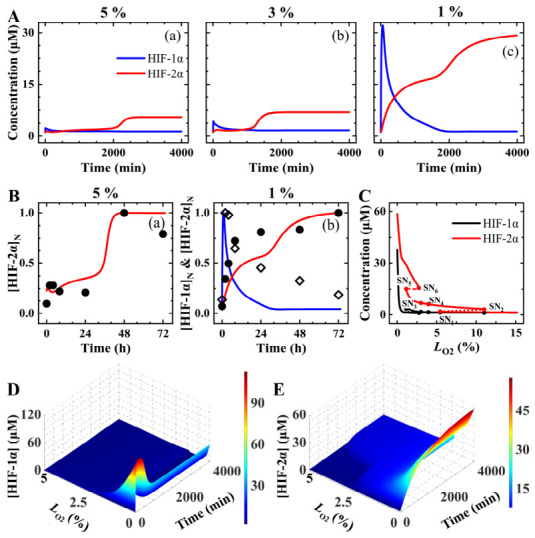
Differential responses of HIF-1α and HIF-2α to hypoxic degree and exposure duration. (**A**) Temporal trajectories of [HIF-1α] (blue) and [HIF-2α] (red) at 5% (**a**), 3% (**b**) and 1% O_2_ (**c**). (**B**) Comparison of model simulations (solid line) with experimental data from SK-N-BE(2)c neuroblastoma cells under 5% O_2_ (**a**) and 1% O_2_ (**b**) [3], where [·]_N_ denotes normalized concentration. (**C**) Bifurcation diagram showing steady-state levels of HIF-1α (black) and HIF-2α (red) as a function of *L*_O2_. (**D**,**E**) Color-coded concentrations of HIF-1α (**D**) and HIF-2α (**E**) as functions of *L*_O2_ and time.

The temporal dynamics of HIF-1α and HIF-2α under varying hypoxia severities reveal their distinct roles in cellular adaptation (Figure 2). HIF-2α gradually accumulates to a plateau under 5% and 3% O_2_ (physioxia and mild hypoxia, respectively), with faster kinetics as hypoxia intensifies (Figure 2A(a,b)). In contrast, HIF-1α exhibits only a modest increase from its basal level in normoxia (21% O_2_) (Figure S1), and its response amplitude remains markedly lower than that of HIF-2α. Under moderate hypoxia (1% O_2_), HIF-1α exhibits a transient response characterized by rapid activation followed by a gradual decline, whereas HIF-2α accumulates continuously to reach a higher plateau (Figure 2A(c)). The kinetic rate of HIF-2α remains slower than that of HIF-1α for *t* < 2000 min. These distinct dynamic profiles reveal a clear temporal division of labor: the early phase of hypoxic response is primarily driven by the rapid activation of HIF-1α, and the late adaptive phase is dominated by HIF-2α, thereby forming the characteristic HIF switch. Notably, the differential responses of HIF-2α and HIF-1α to hypoxia severity and their temporal transition under moderate hypoxia broadly align with experimental observations [3] (Figure 2B(a,b)). It is worth noting that the hypoxia definitions used here differ from the classification of pathological hypoxia: 5% O_2_ as physioxia and 3% O_2_ as mild hypoxia follow the in vitro cell culture standards systematically compiled in Reference [6], whereas 1% O_2_ is defined as moderate hypoxia to distinguish it from the more severe range below 1% O_2_.

The one-parameter bifurcation analysis of [HIF-1α] and [HIF-2α] with respect to oxygen concentration reveals the global evolution of their steady states as *L*_O2_ declines (Figure 2C). Consistent with the trajectory analysis, HIF-2α exhibits a higher oxygen threshold for stabilization and begins to accumulate at ~5% O_2_. This enables HIF-2α to initiate adaptive responses well before the onset of moderate hypoxia (≤2% O_2_), at which HIF-1α becomes markedly stabilized. As *L*_O2_ decreases, the steady-state profiles of [HIF-1α] and [HIF-2α] pass through three regimes separated by successive saddle-node bifurcations (SN_1_–SN_6_), with stepwise increases in expression. Under physioxia, the first bifurcation pair (SN_1_ and SN_2_) drives [HIF-2α] from a low to an intermediate expression, while [HIF-1α] remains on its low-expression branch, indicating the selective early activation of HIF-2α. In the mild hypoxia regime, the second pair (SN_3_ and SN_4_) leaves [HIF-1α] on the low-expression branch and [HIF-2α] on the intermediate branch, suggesting that this transition is insufficient to trigger a pronounced change in either HIF isoform. Under moderate hypoxia, the third pair (SN_5_ and SN_6_) triggers a transition to a higher steady state, leading to simultaneous activation of HIF-1α and HIF-2α and establishing their coordinated expression.

To visualize the dynamics of HIF-1α and HIF-2α over time and across oxygen concentrations, three-dimensional expression landscapes were constructed (Figure 2D,E). As hypoxic exposure progresses from the acute to the chronic phase, HIF-2α gradually stabilizes under prolonged or moderate hypoxia, and its low-expression region (blue) shrinks with increasing exposure time and decreasing oxygen concentration. Although the two HIF isoforms have overlapping expression in the moderate-to-severe hypoxia regime, they exhibit a clear temporal hierarchy: HIF-1α responds rapidly at early stages, whereas HIF-2α predominates later. Overall, these results indicate that HIF-1α and HIF-2α respond differentially to hypoxia intensity and duration, with HIF-2α activated under milder hypoxia and both isoforms coordinately induced under moderate hypoxia.

### 2.2. mTORC1/mTORC2-Dependent Synthesis and PHD-Dependent Degradation Cooperatively Drive the Formation of the HIF Switch

**Figure 3:**
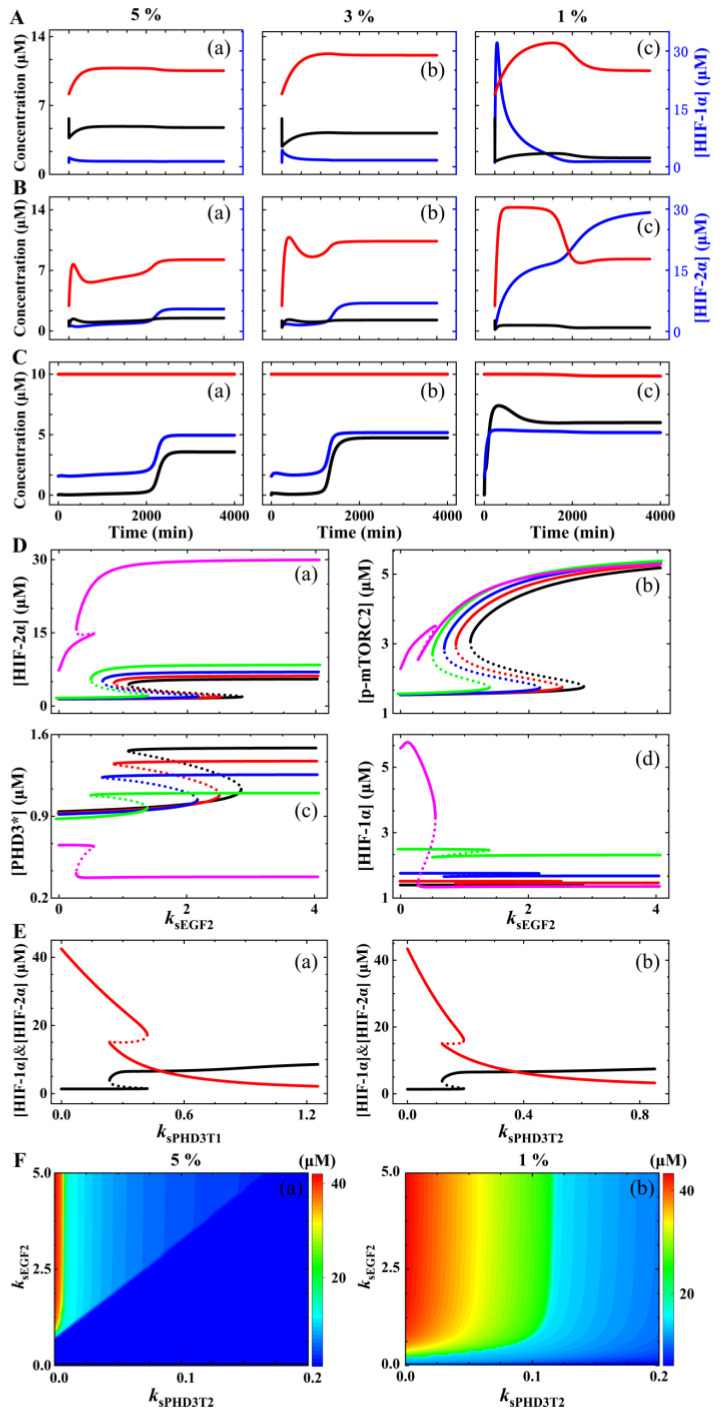
Coordinated regulation of HIF-1α and HIF-2α via both synthesis and degradation. (**A**–**C**) Time courses of [HIF-1α] (blue, referenced to the right *y*-axis), [PHD-2_T_] (red), and [PHD-2*] (black, active form) (**A**), [HIF-2α] (blue, referenced to the right *y*-axis), [PHD-3_T_] (red), and [PHD-3*] (black, active form) (**B**), and [EGF] (black), [mTORC2_T_] (red), and [p-mTORC2] (blue) (**C**) under 5% (**a**), 3% (**b**), and 1% O_2_ (**c**). (**D**) Bifurcation diagrams with respect to *k*_sEGF2_ for [HIF-2α] (**a**), [p-mTORC2] (**b**), [PHD-3*] (**c**), and [HIF-1α] (**d**) at 5% (black), 4% (red), 3% (blue), 2% (green), and 1% (magenta). (**E**) Steady-state levels of [HIF-1α] (black) and [HIF-2α] (red) as functions of *k*_sPHD3T1_ (**a**) and *k*_sPHD3T2_ (**b**) at 1% O_2_. (**F**) Color-coded concentrations of [HIF-2α] as functions of *k*_sPHD3T2_ and *k*_sEGF2_ at 5% (**a**) and 1% O_2_ (**b**).

Here, we investigated the molecular basis of the HIF switch by dissecting the distinct dynamics of HIF-1α and HIF-2α (Figure 3). Consistent with our previous model [34], the pulsatile behavior of HIF-1α at 1% O_2_ arises from negative feedback mediated by PHD-2. Specifically, hypoxia transiently lowers the PHD-2* level, allowing HIF-1α accumulation; accumulated HIF-1α then transcriptionally induces PHD-2, which gradually restores PHD-2* activity and re-establishes suppression of HIF-1α (Figure 3A(c)). Under 5% and 3% O_2_, although the PHD-2* level undergoes a transient dip and recovery, the residual PHD-2* activity remains sufficient to repress HIF-1α, keeping its level close to baseline (Figure 3A(a,b)).

In contrast, the dynamics of HIF-2α cannot be accounted for by a similar degradation-based negative feedback mechanism. Although hypoxia rapidly decreases the PHD-3* level, HIF-2α does not accumulate immediately (Figure 3B(a–c)). Oxygen-dependent degradation alone cannot explain the prolonged low-expression phase (>24 h) that precedes the late upregulation of HIF-2α. Thus, we hypothesized that HIF-2α dynamics are regulated predominantly at the translational level and incorporated a HIF-2α–EGF–PI3K–mTORC2 positive-feedback axis to capture its delayed yet sustained activation.

HIF-2α, EGF, and p-mTORC2 exhibit coordinated late-phase upregulation (Figure 3C(a–c)), indicative of a synthesis-driven positive feedback mechanism. The specifically high sensitivity of the SN_1_ and SN_2_ oxygen thresholds (*L*_SN1_ and *L*_SN2_) to parameters regulating EGF production and mTORC2 activation highlights that this positive feedback governs HIF-2α dynamics in mildly hypoxic regimes (Figure S4). Because activation of positive feedback is inherently threshold-dependent, it naturally introduces pronounced temporal delays; accordingly, the biphasic dynamics of HIF-2α—prolonged low expression followed by a rapid surge—typify canonical positive feedback activation. Taken together, these results suggest that the late-phase upregulation of HIF-2α is governed by this synthesis-level positive feedback loop, a prediction awaiting experimental verification.

To further examine the threshold-dependent activation of this positive feedback, we analyzed the bifurcation of [HIF-2α] with respect to *k*_sEGF2_ (the HIF-2α-dependent EGF production rate). Across oxygen concentrations, both [HIF-2α] and [p-mTORC2] undergo saddle-node bifurcations (Figure 3D(a,b)), confirming that feedback activation necessitates EGF accumulation beyond a critical threshold. Decreasing oxygen concentrations progressively lowers this *k*_sEGF2_ threshold while simultaneously elevating the resulting high-expression plateau. At 1% O_2_, [HIF-2α] reaches a substantially higher plateau than under other conditions (Figure 3D(a)), highlighting the strong regulatory effect of oxygen availability. Mechanistically, the elevation of the [HIF-2α] upper branch correlates with increased [p-mTORC2] (Figure 3D(b)) and, crucially, diminished [PHD-3*] (Figure 3D(c)). Specifically, depletion of PHD-3* at 1% O_2_ relieves HIF-2α degradation and thus drives its pronounced upregulation in this regime.

Given that PHD-3 is a shared target of HIF-1 and HIF-2 and that HIF-2α is markedly elevated under 1% O_2_, HIF-2 is unlikely to induce the low expression of PHD-3. To identify the underlying driver, we analyzed the bifurcation behavior of [HIF-1α] against *k*_sEGF2_ (Figure 3D(d)); HIF-1α undergoes a high-to-low state transition only at 1% O_2_ and decreases sharply as *k*_sEGF2_ increases, indicating that the reduction in PHD-3 is associated with the pronounced decline of HIF-1α, as detailed below. Given the strong influence of PHD-3 on HIF-2α at 1% O_2_, we further constructed bifurcation diagrams of [HIF-2α] against the HIF-1α- and HIF-2α-dependent PHD-3 synthesis rates (*k*_sPHD3T1_ and *k*_sPHD3T2_, respectively; Figure 3E). Enhancing PHD-3 production via either pathway strongly suppresses [HIF-2α] while simultaneously elevating [HIF-1α], highlighting their differential regulation by PHD-3. Furthermore, excessive PHD-3 prevents HIF-2α activation at 1% O_2_, thereby blocking the HIF-1α-to-HIF-2α switch (Figure S2). Collectively, these results establish PHD-3 as a key regulator coordinating the distinct dynamics of HIF-1α and HIF-2α under moderate hypoxia.

Overall, HIF-1α and HIF-2α operate in fundamentally distinct regulatory regimes. While HIF-1α dynamics are dominated by PHD-2-mediated degradation (with relatively constant mTORC1-mediated synthesis), HIF-2α responses to oxygen intensity and exposure time emerge from the combined effects of mTORC2-driven synthesis and PHD-3-mediated degradation. This distinction is further substantiated by a two-parameter bifurcation analysis of [HIF-2α] with respect to *k*_sEGF2_ and *k*_sPHD3T2_ (Figure 3F). Notably, the dominance of these processes shifts with oxygen availability: PHD-3-mediated degradation predominates at 1% O_2_ (Figure 3F(b)), whereas synthesis and degradation are more balanced at 5% O_2_ (Figure 3F(a)). Consistent with this framework, these results are supported by experimental evidence showing that HIF isoforms exhibit distinct synthesis patterns: *HIF-1A* mRNA levels remain largely insensitive to oxygen, whereas *EPAS1* mRNA is robustly upregulated in an oxygen- and time-dependent manner [3].

### 2.3. Fine-Tuned Encoding of OCT4 Expression by Hypoxia Intensity and Duration

**Figure 4:**
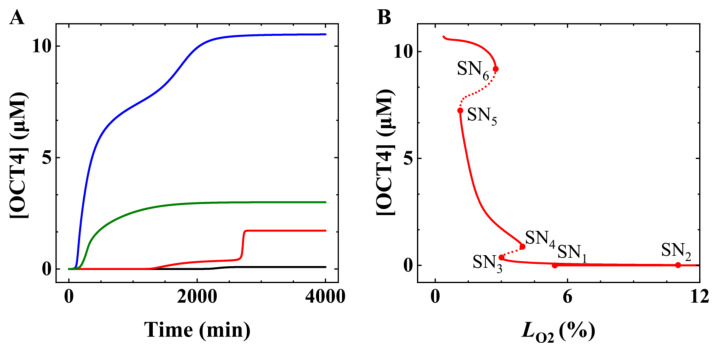
OCT4 expression is tightly regulated by oxygen concentration and exposure time. (**A**) Time courses of [OCT4] under 5% (black), 3% (red), 2% (green), and 1% O_2_ (blue). (**B**) Bifurcation diagram of [OCT4] as a function of *L*_O2_, with stable and unstable steady states denoted by solid and dashed lines, respectively.

Hypoxia promotes tumor malignancy largely by regulating the balance between stemness acquisition and differentiation [37]. Our simulations show that this balance is dynamically tuned by hypoxia severity through its control of stemness factors such as OCT4 (Figure 4).

At 5% O_2_, [OCT4] remains negligible. At 3% and 2% O_2_, [OCT4] gradually reaches intermediate levels, with faster accumulation at 2% O_2_. At 1% O_2_, [OCT4] rapidly rises and finally settles at a high level (Figure 4A). Notably, similar dynamics have been reported experimentally, where OCT4 exhibits gradual accumulation under mild hypoxia but a steep rise under moderate hypoxia [17,38,39]. Building on the dosage-dependent role of OCT4 reported previously [40], this result suggests that mild hypoxia maintains OCT4 at intermediate levels to support stem-like states, whereas moderate hypoxia drives OCT4 to higher levels, thereby promoting differentiation.

The bifurcation diagram of [OCT4] with respect to oxygen concentration *L*_O2_ reveals stepwise transitions across three regimes (Figure 4B), although [OCT4] undergoes three pairs of saddle-node bifurcations, analogous to [HIF-1α] and [HIF-2α] (Figure 2C). The first pair (SN_1_ and SN_2_) lies close to the low-expression branch, inducing little change in [OCT4]. As *L*_O2_ declines to ~3%, [OCT4] traverses SN_3_ of the second bifurcation pair, triggering a transition to the intermediate-expression branch. The onset of moderate hypoxia (~1.1% O_2_) forces a transition past SN_5_ of the third bifurcation pair, driving [OCT4] into the high-expression branch.

Taken together, intermediate and high OCT4 levels are associated with stemness acquisition and differentiation, respectively. Although the first bifurcation pair induces negligible OCT4 expression, it elevates HIF-2α to a moderate level, placing the system in a poised state for stemness acquisition.

### 2.4. HIF-1-Driven Glycolysis and HIF-2-Mediated Transcription Cooperatively Promote OCT4 Expression and Stemness Acquisition

**Figure 5:**
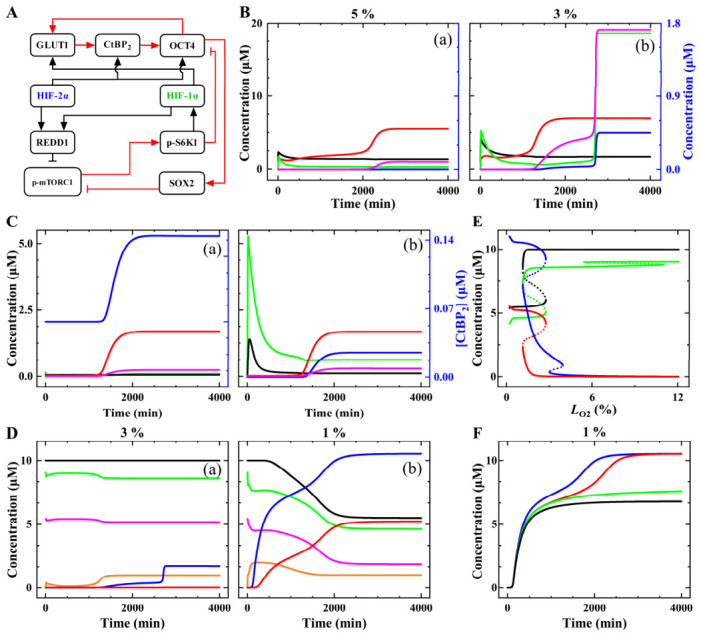
Cooperative regulation of OCT4 expression and stemness by HIF-1 and HIF-2. (**A**) Simplified schematic of the signaling network regulating OCT4 expression. (**B**) Time courses of [HIF-1α] (black), [HIF-2α] (red), [GLUT1] (green), [CtBP_2_] (blue, referenced to the right *y*-axis), and [OCT4] (magenta, referenced to the right *y*-axis) under 5% O_2_ (**a**) and 3% O_2_ (**b**). (**C**) Time courses of [GLUT1] (green), [NADH] (black), [CtBP] (red), [CtBP_2_] (blue, referenced to the right *y*-axis), and [OCT4] (magenta) at *L*_O2_ = 3% under (**a**) *k*_sGLUT1_ = 0 and (**b**) *k*_sGLUT2_ = 0. (**D**) Time courses of [REDD1] (yellow), [mTORC1_T_] (black), [p-mTORC1] (green), [p-S6K1] (magenta), [OCT4] (blue), and [SOX2] (red) at *L*_O2_ = 3% (**a**) and *L*_O2_ = 1% (**b**). (**E**) Bifurcation diagrams of [mTORC1T] (black), [p-mTORC1] (green), [OCT4] (blue), and [SOX2] (red) as a function of *L*_O2_. Stable and unstable steady states are denoted by solid and dashed lines, respectively. (**F**) Time courses of [OCT4] at *L*_O2_ = 1% under four conditions: *k*_sREDD11_ = 0 (red), *k*_sREDD12_ = 0 (green), *k*_sREDD11_ = 0 and *k*_sREDD12_ = 0 (black), and the control (blue).

Because OCT4 expression is tightly dictated by HIF dynamics, we further examined how the HIF switch transduces varying hypoxic signals into distinct OCT4 expression patterns. Topological analysis of the regulatory network reveals that OCT4 is embedded within two coupled positive feedback loops: GLUT1–CtBP_2_–OCT4 and OCT4–SOX2–mTORC1–S6K1 (highlighted in red in Figure 5A). These coupled loops likely underlie the tristable OCT4 dynamics observed above.

To test this possibility and identify the loops responsible for stemness acquisition, we systematically compared the dynamics of key network components at 5% and 3% O_2_. Decreasing oxygen from 5% to 3% elevates OCT4 to an intermediate level, accompanied by marked upregulation of GLUT1 and CtBP_2_ (Figure 5B), whereas the key nodes, including p-mTORC1 and p-S6K1, of the mTORC1 pathway remain largely unchanged (Figure S3). These results suggest that the GLUT1–CtBP_2_–OCT4 loop acts as the primary module driving OCT4 to intermediate levels, thereby mediating stemness acquisition. Notably, *L*_SN3_ and *L*_SN4_ are specifically sensitive to the parameters governing CtBP2 expression, while *L*_SN5_ and *L*_SN6_ exhibit specific sensitivity to the parameters governing OCT4 and SOX2 expression, corroborating that the GLUT1–CtBP2–OCT4 and OCT4–SOX2–mTORC1–S6K1 positive feedback loops, respectively, drive the orderly formation of the second (SN_3_ and SN_4_) and third (SN_5_ and SN_6_) bifurcation pairs.

Notably, this feedback loop is regulated by both HIF-1α and HIF-2α. HIF-2α functions as a key activator of OCT4 transcription to drive stemness acquisition, whereas the specific contribution of HIF-1α remains to be clarified. To this end, we selectively blocked the HIF-1- or OCT4-dependent GLUT1 synthesis (*k*_sREDD11_ = 0 or *k*_sREDD12_ = 0) at 3% O_2_ (Figure 5C). For *k*_sREDD11_ = 0, GLUT1, NADH, CtBP, and OCT4 all remain at low levels (Figure 5C(a)). Conversely, blocking OCT4-dependent GLUT1 synthesis triggers a transient elevation in GLUT1 and NADH followed by a subsequent decline, while CtBP_2_ and OCT4 remain persistently suppressed (Figure 5C(b)). These results indicate that HIF-1α initiates glycolysis by inducing GLUT1 and related glycolytic factors, thereby priming the GLUT1–CtBP_2_–OCT4 loop. Subsequent coupling between metabolic activation and OCT4 regulation then drives the full activation of this loop, enabling the acquisition and maintenance of cellular stemness. Consistent with previous studies showing that glycolysis supports stemness maintenance [41,42], the cooperation between HIF-1α-mediated glycolytic priming and HIF-2α-dependent OCT4 induction likely represents a central mechanism by which the HIF switch coordinates stemness acquisition.

### 2.5. Inhibition of Protein Synthesis by the REDD1–mTORC1 Axis Drives High OCT4 Expression and Cellular Differentiation

We further examined the role for the OCT4–SOX2–mTORC1–S6K1 positive feedback loop in driving OCT4 from an intermediate to a high level. Comparison of the network dynamics at 3% and 1% O_2_ reveals distinct regulatory shifts (Figure 5D). While SOX2, mTORC1T, p-mTORC1, and p-S6K1 change only modestly at 3% O_2_ (Figure 5D(a)), OCT4 increases sharply at 1% O_2_, accompanied by strong SOX2 upregulation and a concomitant, marked decline in mTORC1_T_, p-mTORC1, and p-S6K1 (Figure 5D(b)). These coordinated changes suggest that activation of the OCT4–SOX2–mTORC1–S6K1 loop facilitates the transition of [OCT4] to a high-expression plateau.

Consistently, bifurcation analysis of [mTORC1_T_], [p-mTORC1], [SOX2], and [OCT4] with respect to *L*_O2_ reveals a pronounced state transition around 1.1% O_2_: the former two shift to lower steady states, whereas the latter two transition to higher steady states (Figure 5E). Thus, OCT4 upregulation at 1% O_2_ is closely associated with global repression of protein synthesis. Under severe energy constraints, cells suppress energetically demanding translation while selectively enhancing the expression of key fate regulators, thereby facilitating adaptive cell-fate reprogramming. In this state, cooperative induction of angiogenesis-related factors such as VEGF by HIF-1 and HIF-2 may further bias tumor cells toward a vascular-lineage phenotype [43].

Previous studies have shown that hypoxia-induced inhibition of mTORC1 signaling can be mediated by HIF-1α- and HIF-2α-dependent induction of REDD1. To assess the role of REDD1 in OCT4 upregulation, we selectively blocked HIF-1-dependent REDD1 synthesis (*k*_sREDD11_ = 0), HIF-2-dependent REDD1 synthesis (*k*_sREDD12_ = 0), or both simultaneously (Figure 5F). Compared with the control, blocking only HIF-1-dependent REDD1 synthesis merely delays the onset of the OCT4 surge without altering its final amplitude. In contrast, abrogating HIF-2-dependent REDD1 synthesis, either alone or alongside the HIF-1 pathway, traps OCT4 at an intermediate level and prevents its transition to the high-expression state. Thus, HIF-1α primarily regulates the timing of OCT4 upregulation, whereas HIF-2α determines whether OCT4 can reach the high-expression state and acts as the principal driver of differentiation.

## 3. Discussion

Long-term hypoxia is a critical driver of physiological processes, such as angiogenesis and skeletal formation during embryonic development [6], and a key regulator of tumor cell adaptation and stemness [25]. Importantly, prolonged hypoxia induces a temporal transition from HIF-1 to HIF-2 signaling, known as the “HIF switch”, which has been implicated in stemness regulation [6]. However, the underlying regulatory mechanisms remain poorly understood.

**Figure 6:**
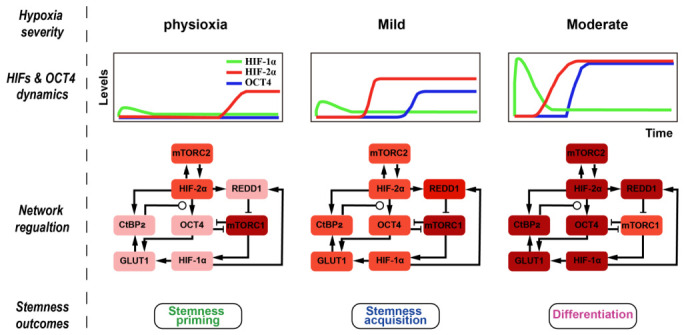
Worsening hypoxia dynamically rewires the HIF-1/HIF-2 regulatory network, driving the HIF switch and orchestrating stage-specific stemness transitions.

To address this, we developed a network model that integrates the HIF-1/HIF-2 axis, mTORC1/2 signaling, and the core stemness regulators OCT4 and SOX2. Our results demonstrate that graded hypoxia differentially modulates HIF-1α, HIF-2α, and OCT4 levels, thereby orchestrating a sequential transition of cellular states: from a primed state under physioxia, to a stem-like state under mild hypoxia, and ultimately to a differentiated state under moderate hypoxia (Figure 6). Mechanistically, this transition is governed by the coordinated activation of interconnected modules, including differential HIF synthesis, glycolysis–stemness crosstalk, and stemness–protein synthesis coupling (Figure 6).

We propose that the HIF switch under moderate hypoxia emerges from the coordination of two coupled circuits: the HIF-1α–PHD-2 negative feedback and HIF-2α–EGF–PI3K–mTORC2 positive feedback loops. The former drives pulsatile HIF-1α dynamics (rapid accumulation followed by progressive attenuation), whereas the latter enables threshold-dependent, switch-like activation of HIF-2α. This architecture reveals a fundamental regulatory asymmetry: HIF-1α dynamics are primarily governed by oxygen-dependent degradation, whereas HIF-2α dynamics are dominated by synthesis-driven regulation, with degradation playing only a permissive role. Supporting this, experimental data show stable or slightly declining HIF-1α mRNA [3,19], alongside a pronounced, time-dependent increase in HIF-2α mRNA during prolonged hypoxia [3].

Although our model reveals a core dynamical mechanism for the HIF switch, it does not exclude synergy with other regulatory layers. For example, hypoxia-associated factor (HAF) may facilitate this transition by concurrently promoting HIF-1α degradation and enhancing HIF-2α transcriptional activity [44], although its own regulation remains unclear [45]. Similarly, the Hsp70/CHIP complex selectively ubiquitinates HIF-1α and promotes its degradation under chronic hypoxia, thereby facilitating the HIF switch [46]. Overall, these findings show that the HIF switch is governed by multiple layers of regulation, including protein stability, translational control, protein stability, and transcription, across distinct hierarchies. How they are integrated at the systems level to shape HIF-1α decay and delayed HIF-2α accumulation remains an open question.

The mechanisms for balancing hypoxia-induced stemness and differentiation remain elusive, particularly given the context-dependent role of HIF-1α [47]. We propose a unified regulatory framework where hypoxia severity dictates cell fate through stage-dependent HIF-1/2 coordination. Under physioxia (~5% O_2_), HIF-2α accumulates but remains sub-threshold, leaving cells in a primed, non-committed state. Under mild hypoxia (~2% O_2_), HIF-1α-driven glycolysis activates the NADH–CtBP–HIF-2α–OCT4 axis, driving stemness acquisition through metabolic-transcriptional coupling. Under moderate hypoxia (~1% O_2_), REDD1 induction, mediated by both HIF subunits, suppresses mTORC1–S6K1 signaling, releasing OCT4 from inhibition. This drives OCT4 to a high-expression plateau, promoting differentiation (e.g., toward vascular lineages) [48]. In this framework, HIF-1α acts as a functional switch, supporting stemness via metabolism under mild hypoxia, but favoring differentiation under moderate hypoxia. This transition is further reinforced by global protein synthesis suppression at 1% O_2_ [49], which biases cells toward differentiation rather than energetically demanding stem-like states.

Elucidating the molecular mechanisms by which the HIF switch regulates glycolysis-dependent stemness acquisition is critical for understanding hypoxia-driven tumor cell plasticity. Glycolysis is widely recognized as a key metabolic foundation for stemness acquisition [15,25,50]; however, it is primarily governed by HIF-1α rather than HIF-2α, which is more closely associated with stemness regulation [8,25]. This functional specialization suggests that HIF-1α and HIF-2α may cooperatively regulate the glycolysis–stemness axis through temporally distinct mechanisms. As suggested by our model, the HIF switch-mediated transition from HIF-1α to HIF-2α provides a dynamic link between metabolic reprogramming and activation of the stemness program. During early hypoxia, rapid HIF-1α activation enhances glycolytic flux, thereby providing the energetic and biosynthetic support required for hypoxic adaptation and the initiation of stemness-associated programs. As hypoxia persists, progressive HIF-2α accumulation stabilizes core stemness regulators such as OCT4 [8], thereby converting the early metabolic adaptation initiated by HIF-1α into a sustained stemness program.

Furthermore, we propose a cooperative metabolic-stemness axis centered on the OCT4–GLUT1 positive feedback loop, which stabilizes OCT4 at intermediate levels to maintain the stem-like state. Beyond this circuit, glycolysis regulates stemness through multiple mechanisms: glycolytic intermediates modulate epigenetic states [51], while lactate can alter intracellular pH to enhance WNT/β-catenin signaling [52]. Additionally, glycolytic enzymes may directly interact with stemness-regulatory proteins [53]. A systematic elucidation of these metabolic, epigenetic, and signaling interdependencies is essential to fully decode the hypoxia–metabolic reprogramming–cell fate regulatory network.

In addition to OCT4 and SOX2, other stemness-associated factors, including NANOG, KLF4, and c-MYC, may also participate in hypoxia-induced stemness regulation [54,55]. Notably, the proximal promoter region of NANOG contains Oct–Sox cis-regulatory elements [56], suggesting that HIF-2α-mediated regulation of NANOG may occur primarily through OCT4/SOX2. Thus, NANOG may exhibit multi-stable dynamics similar to those observed for OCT4. OCT4, SOX2, and NANOG are well-established core pluripotency transcription factors that mutually reinforce one another and form a highly coupled positive-feedback network [57]. This regulatory motif is essential for maintaining pluripotency and self-renewal in embryonic stem cells [58]. However, whether this core pluripotency network contributes to hypoxia-induced acquisition of stemness remains to be determined. One possible hypothesis is that hypoxia first promotes stemness-associated features through the OCT4–SOX2 loop, after which activated NANOG further maintains the stem-like state. Moreover, NANOG overexpression can promote cell proliferation by binding to regulatory regions of key cell-cycle genes, such as CDK6 and CDC25A, suggesting crosstalk between stemness regulation and cell-cycle control [17]. Notably, NANOG expression is highly heterogeneous, varying not only across cells within the same population but also dynamically over time at the single-cell level [59,60]. This heterogeneity may reflect the context- and cell-state-dependent role of NANOG in stemness regulation.

Integrating our current and previous findings establishes a unified two-dimensional framework, spanning time and intensity, to explain hypoxic adaptation. While our earlier work focused on acute hypoxia and the sequential activation of adaptive programs via HIF-1α hydroxylation states [33,36], the present study reveals that under chronic, graded hypoxia, cell fate is determined by a specialized division of labor within the HIF network. Specifically, physioxia facilitates a stemness-primed state via delayed HIF-2α accumulation, which progresses to stemness acquisition at mild levels as HIF-1α-driven glycolysis provides the necessary metabolic support. As hypoxia advances to moderate levels, dual HIF activation and suppressed protein synthesis bias cells toward differentiation (e.g., VEGF-driven angiogenesis), while severe hypoxia triggers p53-mediated apoptosis or necrosis [34]. This functional specialization across hypoxic gradients provides a comprehensive logic for cellular decision-making, though the persistent expression of HIF-2α at extreme hypoxia remains a conceptual inconsistency that warrants further investigation.

One important application of the model is that it can serve as an extensible foundational network module for integrating multilevel regulators of hypoxic responses and cell fate decisions in pathophysiological hypoxic environments, thereby better explaining complex cellular responses in real biological contexts. For example, the energy sensor AMPK could be incorporated to link cellular energy stress with glycolytic metabolism, mTORC1-dependent protein synthesis, and ULK1-mediated autophagy, thereby connecting energy sensing, proteostasis, mitochondrial homeostasis, and cellular stemness [61,62]. Under prolonged hypoxia, insufficient energy supply and reduced protein synthesis may shift cells from stemness maintenance toward differentiation or other stress-associated fates. ROS homeostasis may provide another regulatory layer linking hypoxic metabolic adaptation to stemness control [18]. Hypoxia can reshape ROS levels by altering mitochondrial electron transport, glycolytic dependence, and antioxidant systems such as NADPH/glutathione. Moderate ROS may function as signaling molecules regulating HIF stability, AMPK/mTOR signaling, autophagy, and stemness-associated pathways, whereas excessive or sustained ROS may promote oxidative damage, differentiation, or cell death [15,18]. In pathophysiological hypoxic environments, cell fate is also shaped by inflammatory, paracrine, and extracellular microenvironmental inputs. IL-6–JAK/STAT3 signaling, for instance, could be incorporated as an inflammatory input that regulates stemness-associated transcription factors such as OCT4, SOX2, and NANOG, while also interacting with HIF signaling through effects on HIF-1α stability or hypoxia-related transcriptional programs [63]. Similarly, TGF-β/EMT-related modules could be integrated to examine whether the HIF switch cooperates with EMT state transitions to regulate stemness, differentiation, and invasive capacity under prolonged hypoxia [35,64]. Cell-cycle-dependent dormancy represents another important future extension. Prolonged hypoxia, energy stress, and reduced protein synthesis may promote cell-cycle exit and drive cells into low-proliferative or dormancy-like states. This could be modeled by adding an mTORC1/S6K1-dependent cell-cycle module, such as the Cyclin D/E–CDK2/4/6–Rb–E2F axis, together with the HIF-1α–p21/p27 axis for hypoxia-induced cell-cycle inhibition [65,66]. Such an extension would allow the model to distinguish among stemness maintenance with proliferative potential, dormancy, and dormant stem-like states under hypoxia.

Several key predictions made by the model can be experimentally tested. HIF-2α is predicted to exhibit a delayed increase driven by the HIF-2α–EGF–PI3K–mTORC2 positive feedback loop, which, together with the single-pulse dynamics of HIF-1α under HIF-1α–PHD2 negative feedback, generates a HIF switch. Following the strategy of Bagnall et al., HIF-1α and HIF-2α can be simultaneously monitored using distinct fluorescent fusion proteins (e.g., HIF-1α–EGFP and HIF-2α–mCherry) with time-lapse confocal microscopy to capture both the graded HIF-2α response and the fine dynamics of the HIF switch [32]. To assess the contribution of positive feedback to HIF-2α kinetics, cells can be treated with cycloheximide (translation inhibition) or MG132 (proteasome inhibition) at different time points to distinguish the contributions of protein synthesis and degradation, with fluorescence distributions analyzed by flow cytometry to detect bimodal patterns. PHD3’s role in regulating HIF-2α dynamics can be investigated using PHD3 knockout and overexpression cell lines [67]. Moderate hypoxia (e.g., 1% O_2_) triggers cellular differentiation by reducing global protein synthesis, which can be validated by determining whether the decline in protein synthesis precedes differentiation and by experimentally modulating synthesis levels to confirm their causal role in maintaining stemness. Regulation of cellular stemness according to OCT4 expression levels under hypoxia can be assessed by exposing cells to prolonged hypoxic conditions with varying oxygen levels (e.g., 3% or 1% oxygen) and analyzing them by single-cell RNA sequencing (scRNA-seq) with pseudotime analysis. This approach allows identification of cell subpopulations with high OCT4 expression and assessment of their enrichment for differentiation-associated pathways, including angiogenesis, vascular development, and endothelial differentiation [68]. Notably, renal cell carcinoma may serve as a candidate system for integrative validation of the HIF switch and its regulatory effects on cellular stemness, as tumor progression in RCC has been associated with a gradual shift from HIF-1α-dominant to HIF-2α-dominant signaling [6].

While our model captures the coordinated roles of HIF-1 and HIF-2, several limitations offer avenues for future refinement. First, the model focuses on a binary switch, whereas emerging evidence suggests a more complex triphasic sequence (HIF-1 to HIF-2 to HIF-3) that remains to be fully characterized [69]. Second, the simulated HIF-1α decay is faster than experimental observations, indicating that additional regulatory processes beyond PHD-2-mediated feedback likely shape its in vivo dynamics. Third, integrating differentiation-associated modules, such as VEGF-driven angiogenesis, would extend the current framework into a truly unified model of hypoxia-induced cell fate. Finally, incorporating stochastic fluctuations and energy landscape frameworks could provide deeper insights into how biological noise influences the systems-level stability of the HIF switch [70].

In summary, the HIF dual-isoform network model provides a quantitative, systems-level framework for dissecting the molecular logic of the HIF switch and its coupling to metabolic reprogramming and stemness regulation. Future incorporation of HIF-3α, differentiation-associated modules, and the mechanisms governing HIF-2α under extreme hypoxia will enable the construction of a multi-layered architecture spanning both temporal scales and hypoxic intensities. Such an integrated framework provides a conceptual foundation for understanding how hypoxia orchestrates cell-fate decision to drive tumor progression.

## 4. Materials and Methods

### 4.1. Hypoxia Sensing

In the model, oxygen sensing is governed by the oxygen-dependent hydroxylase activity of PHDs [10]. Among the three major isoforms (PHD-1, PHD-2, PHD-3), only PHD-2 and PHD-3 are considered as the primary regulators of the HIF switch. PHD1 was not included because its hypoxia-induced expression is generally weaker and more context-dependent than that of PHD2 and PHD3 [71]. In contrast, PHD-2 and PHD-3 exhibit distinct, hypoxia-responsive roles: PHD-2 is primarily induced by HIF-1α, whereas PHD-3 is cooperatively regulated by both HIF-1α and HIF-2α [71]. These isoforms also display differential substrate preferences: PHD-2 mainly hydroxylates HIF-1α under normoxia, while PHD-3 becomes the dominant regulator of HIF-2α stability under prolonged hypoxic conditions [72].

Each PHD isoform is represented by two forms: an active (PHD*) and an inactive (PHD) form, whose interconversion is regulated by oxygen concentration (see Equations (S3) and (S7) in the Supplementary Materials). Their total abundances are denoted by PHD-2_T_ and PHD3_T_, respectively. HIF-1α induces PHD-2 production through a Hill-type regulatory function (Equation (S2)), whereas PHD-2* primarily inhibits HIF-1α (Equation (S1)), forming a negative feedback loop. In parallel, HIF-1α and HIF-2α cooperatively induce PHD-3 expression via a Hill-type function (Equation (S6)), while PHD-3* mainly suppresses HIF-2*α* (Equation (S5)). Together, these interactions define two core negative feedback loops: the HIF-1α–PHD-2 and HIF-2α–PHD-3 loops.

### 4.2. Regulation of HIF Synthesis

The synthesis of HIF-α is controlled by the growth factor-dependent PI3K–AKT–mTOR pathway [73,74]. Upon growth factor stimulation, PI3K activation promotes the initial phosphorylation of AKT at Thr308, followed by mTORC2-mediated phosphorylation at Ser473, leading to full AKT activation [75]. Activated AKT inhibits the tuberous sclerosis complex (TSC1/TSC2), thereby relieving its suppression of mammalian target of rapamycin complex 1 (mTORC1). As a central regulator of cellular anabolic metabolism, activated mTORC1 enhances ribosome biogenesis and protein translation through phosphorylation of its downstream effector p70 ribosomal protein S6 kinase 1 (S6K1). This signaling cascade constitutes the principal mechanism by which growth factor signaling promotes the rapid accumulation of HIF-1α [76]. In contrast, the synthesis of HIF-2α is highly dependent on mTORC2 activity [73,74].

Both HIF-1α and HIF-2α promote EGF expression, which activates the PI3K pathway to establish HIF-1α–EGF–PI3K–AKT–mTORC1–S6K1 and HIF-2α–EGF–PI3K–mTORC2 positive feedback loops, thereby fine-tuning HIF-1α and HIF-2α synthesis [21]. In addition, stabilized HIF induces regulated in development and DNA damage responses 1 (REDD1) expression, which activates the TSC1/TSC2 complex to suppress mTORC1 activity and reduce HIF-1α synthesis, thereby introducing negative feedback to limit excessive HIF-1α accumulation [77]. In contrast, the upstream mTORC2 pathway is exempt from REDD1-mediated inhibition and thus does not exert negative feedback on HIF-2α.

The PI3K, AKT, mTORC1, mTORC2, and S6K1 kinases are each represented by an active phosphorylated form and an inactive non-phosphorylated form. The total abundances of PI3K, AKT, and S6K1 are assumed to be constant. All enzyme-catalyzed reactions in this module are described using Michaelis–Menten kinetics (Equations (S11)–(S22)).

### 4.3. HIF-Mediated Metabolic Reprogramming and Metabolic Sensing

Hypoxia-induced metabolic reprogramming is represented in the model by the HIF-1-driven shift from oxidative phosphorylation to glycolysis [8]. Mechanistically, HIF-1 activates glycolytic and glucose uptake genes, including GLUT1 and key rate-limiting enzymes, and induces pyruvate dehydrogenase kinase (PDK), thereby inhibiting pyruvate entry into the tricarboxylic acid (TCA) cycle. Consequently, metabolic flux is redirected toward cytosolic glycolysis, consistent with the metabolic phenotype of hypoxic cells [8,78].

This metabolic shift is coupled to redox remodeling through changes in the NADH/NAD^+^ balance. Under normoxia, NADH is continuously oxidized to NAD^+^ through mitochondrial respiration. Under hypoxia, reduced oxidative capacity limits this process and favors glycolysis-dependent metabolism, leading to an increase in the intracellular NADH/NAD^+^ ratio [79,80]. Elevated NADH then promotes CtBP dimerization, converting monomeric CtBP into its active dimeric form, CtBP_2_, which mediates downstream transcriptional regulation [81]. In addition, HIF-2α directly promotes CtBP transcription [28]. Thus, HIF-1 primarily controls metabolic flux toward NADH production, whereas HIF-2α contributes to CtBP expression.

Here, GLUT1 is selected as a representative node of HIF-1-mediated glycolytic regulation, and its production rate is described by a Hill-type function (Equation (S23)). GLUT1 mediates the transmembrane transport of extracellular glucose (Glucose_out_) into the intracellular pool (Glucose_in_) (Equation (S24)). Increased intracellular glucose drives NADH production, which in turn regulates the conversion between monomeric CtBP and dimeric CtBP_2_ (Equations (S25)–(S28)).

### 4.4. Hypoxia-Mediated Acquisition of Stemness

Hypoxia-induced acquisition of stem-like traits is incorporated in the model through the HIF-2α–CtBP_2_–OCT4 module. HIF-2α directly activates OCT4 transcription by binding to its proximal promoter [56], and this activation is further amplified by CtBP_2_. Mechanistically, CtBP_2_ interacts with PXDLS motif-containing transcription factors, including HIF-2α, and acts as a scaffold for coactivator complexes, facilitating the recruitment of chromatin-modifying enzymes and additional cofactors [28]. In turn, OCT4 can upregulate GLUT1 expression [29], thereby enhancing glucose uptake and glycolytic flux, increasing NADH production, and activating CtBP. These interactions form a positive feedback loop, GLUT1–Glucose–NADH–CtBP–OCT4, that stabilizes OCT4 expression and supports stemness under hypoxia.

This module is further coupled to mTOR signaling through the OCT4-SOX2-mTORC1-S6K1 axis. Moderate levels of OCT4 enhance the transcriptional activity of SOX2 [82], which in turn suppresses both mTORC1 and mTORC2 by mediating nucleosome remodeling and recruiting the nucleosome remodeling and deacetylase (NuRD) complex to the mTOR promoter [83]. S6K1 negatively regulates OCT4 stability by promoting its phosphorylation and subsequent proteasomal degradation [84]. Together, these interactions provide a homeostatic mechanism for stemness maintenance.

Here, cooperative activation of OCT4 by HIF-2α and CtBP_2_, as well as the transcriptional regulation of SOX2 by OCT4, is modeled using Hill-type functions (Equations (S29) and (S30)). The inhibitory effects of SOX2 on mTORC1 and mTORC2 expression are represented by SOX2-dependent inhibitory Hill functions for mTORC1_T_ and mTORC2_T_, respectively (Equations (S15) and (S20)). The destabilizing effect of p-S6K1 on OCT4 is included in the OCT4 degradation term and modeled using Michaelis–Menten kinetics (Equation (S29)).

Together, our network model comprises 30 nodes, with hypoxia severity as the input and stemness, represented by OCT4 levels, as the sole output. All regulatory interactions among the nodes were curated from previously published studies. Each molecular species is described by a state variable, and the system dynamics are governed by 22 ordinary differential equations (ODEs) detailed in the Supporting Information (Supplementary Method S1). [·] denotes the concentration of species. A subset of model parameters was obtained from published studies, whereas parameters without directly available literature values were estimated within biologically plausible ranges. These estimates were constrained by the ability of the model to reproduce experimentally reported HIF switch dynamics, such as those observed in SK-N-BE neuroblastoma cells [3], and to ensure that all nodes follow experimentally supported response trends under hypoxia. All parameter values are listed in Supplementary Table S2. The oxygen level (*L*_O2_) is defined as the percentage of oxygen volume in air. Initial conditions for all variables, as listed in Supplementary Table S1, were set to their lower steady-state values under normoxia (i.e., *L*_O2_ = 21%). All ODEs were numerically solved using Oscill8, which was also employed to perform single- and two-parameter bifurcation analyses. Time is in units of minutes, concentration is in μM, and other parameter units were set such that all state variables remain dimensionless.

## Data Availability

The original contributions presented in this study are included in the article/Supplementary Material. Further inquiries can be directed to the corresponding authors.

## Supplementary Materials

The following supporting information can be downloaded at: https://www.mdpi.com/article/10.3390/ijms27114697/s1. References [85,86,87,88] are cited in the supplementary materials.

## Abbreviations

The following abbreviations are used in this manuscript:

HIF-1α	Hypoxia-inducible factor 1α
HIF-2α	Hypoxia-inducible factor 2α
PHD-1	Prolyl hydroxylase domain-containing protein 1
PHD-2	Prolyl hydroxylase domain-containing protein 2
PHD-3	Prolyl hydroxylase domain-containing protein 3
OCT4	Octamer-binding transcription factor 4
REDD1	Regulated in development and DNA damage responses 1
mTORC1	Mechanistic target of rapamycin complex 1
mTORC2	Mechanistic target of rapamycin complex 2
EGF	Epidermal growth factor
PI3K	Phosphatidylinositol 3-kinase
AKT	Protein kinase B
S6K1	Ribosomal protein S6 kinase beta-1
GLUT1	Glucose transporter 1
CtBP	C-terminal binding protein
SOX2	SRY-box transcription factor 2
NADH	Nicotinamide adenine dinucleotide (reduced form)
NAD^+^	Nicotinamide adenine dinucleotide (oxidized form)
