# Supplementary material for: A Mechanistic Model of the HIF-1/HIF-2 Switch Regulating Hypoxia-Induced Cancer Stemness
Source: Int J Mol Sci. 2026 May 23;27(11):4697. doi: 10.3390/ijms27114697 (PMC13256912; doi:10.3390/ijms27114697)
Supplement: Supplementary file 1 [file ijms-27-04697-s001.zip › ijms-4292636-supplementary.pdf]

SUPPORTING MATERIAL

A Mechanistic Model of the HIF-1/HIF-2 Switch Regulating Hypoxia-Induced Cancer Stemness

In the present study, we constructed a network model to elucidate the molecular mechanism underlying the HIF switch and its regulatory role in cancer cell stemness. The involved ordinary differential equations, initial values, and parameter values were listed here. The initial values were set to the steady-state value of each variable at 21% O_2.

Supplemental Method S1: Equations of the model

$$\frac{d[\text{HIF-1}\alpha]}{dt} = k_{\text{sHIF1}\alpha}[\text{p-S6K1}] - k_{\text{deHIF1}\alpha}[\text{PHD-2}^*]\frac{[\text{HIF-1}\alpha]}{[\text{HIF-1}\alpha]+j_{\text{deHIF1}\alpha}} - d_{\text{HIF1}\alpha}[\text{HIF-1}\alpha] \quad \text{(S1)}$$

$$\frac{d[\text{PHD-2}_\text{T}]}{dt} = k_{\text{sPHD2T0}} + k_{\text{sPHD2T1}}\frac{[\text{HIF-1}\alpha]^4}{[\text{HIF-1}\alpha]^4+{j_{\text{sPHD2T1}}}^4} - d_{\text{PHD2T}}[\text{PHD-2}_\text{T}] \quad \text{(S2)}$$

$$\frac{d[\text{PHD-2}^*]}{dt} = k_{\text{acPHD2}}\frac{\text{ss}*\text{O}_2}{\text{ss}*\text{O}_2+j_{\text{O2PHD2}}}[\text{PHD-2}] - k_{\text{dePHD2}}[\text{PHD-2}^*] \quad \text{(S3)}$$

$$[\text{PHD-2}] = [\text{PHD-2}_\text{T}] - [\text{PHD-2}^*] \quad \text{(S4)}$$

$$\frac{d[\text{HIF-2}\alpha]}{dt} = k_{\text{sHIF2}\alpha}\frac{[\text{p-mTORC2}]^4}{[\text{p-mTORC2}]^4+{j_{\text{sHIF2}\alpha}}^4} - k_{\text{deHIF2}\alpha}\frac{[\text{PHD-3}^*][\text{HIF-2}\alpha]}{[\text{HIF-2}\alpha]+j_{\text{deHIF2}\alpha}} - d_{\text{HIF2}\alpha}[\text{HIF-2}\alpha] \quad \text{(S5)}$$

$$\frac{d[\text{PHD-3}_\text{T}]}{dt} = k_{\text{sPHD3T0}} + k_{\text{sPHD3T1}}\frac{[\text{HIF-1}\alpha]^4}{[\text{HIF-1}\alpha]^4+{j_{\text{sPHD3T1}}}^4} + k_{\text{sPHD3T2}}\frac{[\text{HIF-2}\alpha]^4}{[\text{HIF-2}\alpha]^4+{j_{\text{sPHD3T2}}}^4} - d_{\text{PHD3T}}[\text{PHD-3}_\text{T}] \quad \text{(S6)}$$

$$\frac{d[\text{PHD-3}^*]}{dt} = k_{\text{acPHD3}}\frac{\text{ss}*\text{O}_2}{\text{ss}*\text{O}_2+j_{\text{O2PHD3}}}[\text{PHD-3}] - k_{\text{dePHD3}}[\text{PHD-3}^*] \quad \text{(S7)}$$

$$[\text{PHD-3}] = [\text{PHD-3}_\text{T}] - [\text{PHD-3}^*] \quad \text{(S8)}$$

$$\frac{d[\text{REDD1}]}{dt} = k_{\text{sREDD10}} + k_{\text{sREDD11}}\frac{[\text{HIF-1}\alpha]^4}{[\text{HIF-1}\alpha]^4+{j_{\text{sREDD11}}}^4} + k_{\text{sREDD12}}\frac{[\text{HIF-2}\alpha]^4}{[\text{HIF-2}\alpha]^4+{j_{\text{sREDD12}}}^4} - d_{\text{REDD1}}[\text{REDD1}] \quad \text{(S9)}$$

$$\frac{d[\text{EGF}]}{dt} = k_{\text{sEGF0}} + k_{\text{sEGF1}}\frac{[\text{HIF-1}\alpha]^4}{[\text{HIF-1}\alpha]^4+{j_{\text{sEGF1}}}^4} + k_{\text{sEGF2}}\frac{[\text{HIF-2}\alpha]^4}{[\text{HIF-2}\alpha]^4+{j_{\text{sEGF2}}}^4} - d_{\text{EGF}}[\text{EGF}] \quad \text{(S10)}$$

$$\frac{d[\text{p-PI3K}]}{dt} = k_{\text{sPI3K}}\frac{\text{EGF}+\text{GF}_0}{\text{EGF}+\text{GF}_0+j_{\text{GF}}}\frac{[\text{PI3K}]}{[\text{PI3K}]+j_{\text{PI3K}}} - d_{\text{PI3K}}[\text{p-PI3K}] \quad \text{(S11)}$$

$$[\text{PI3K}] = [\text{PI3K}_\text{T}] - [\text{p-PI3K}] \quad \text{(S12)}$$

$$\frac{d[\text{p-AKT}]}{dt} = \left(k_{\text{acAKT}} + k_{\text{acAKT1}}\frac{[\text{p-mTORC2}]}{[\text{p-mTORC2}]+j_{\text{pAKT1}}}\right)\frac{[\text{p-PI3K}]}{[\text{p-PI3K}]+j_{\text{pAKT2}}}\frac{[\text{AKT}]}{[\text{AKT}]+j_{\text{acAKT}}} - k_{\text{deAKT}}\frac{[\text{p-AKT}]}{[\text{p-AKT}]+j_{\text{deAKT}}} \quad \text{(S13)}$$

$$[\text{AKT}] = [\text{AKT}_\text{T}] - [\text{p-AKT}] \quad \text{(S14)}$$

$$\frac{d[\text{mTORC1}_\text{T}]}{dt} = k_{\text{smTORC1T1}} + k_{\text{smTORC1T2}}\frac{{j_{\text{smTORC1T2}}}^4}{[\text{Sox2}]^4+{j_{\text{smTORC1T2}}}^4} - d_{\text{mTORC1T}}[\text{mTORC1}_\text{T}] \quad \text{(S15)}$$

$$\frac{d[\text{p-mTORC1}]}{dt} = k_{\text{acmTORC1}}\frac{[\text{p-AKT}]}{[\text{p-AKT}]+j_{\text{pmTORC1}}}\frac{[\text{mTORC1}]}{[\text{mTORC1}]+j_{\text{acmTORC1}}}$$
$$-k_{\text{depmTORC11}}\frac{[\text{p-mTORC1}]}{[\text{p-mTORC1}]+j_{\text{dpmTORC1}}} - k_{\text{depmTORC12}}[\text{REDD1}]\frac{[\text{p-mTORC1}]}{[\text{p-mTORC1}]+j_{\text{depmTORC1}}} \quad \text{(S16)}$$

$$[\text{mTORC1}] = [\text{mTORC1}_\text{T}] - [\text{p-mTORC1}] \quad \text{(S17)}$$

$$\frac{d[\text{p-S6K1}]}{dt} = k_{\text{acS6K1}}[\text{p-mTORC1}]\frac{[\text{S6K1}]}{[\text{S6K1}]+j_{\text{acS6K1}}} - k_{\text{depS6K1}}\frac{[\text{p-S6K1}]}{[\text{p-S6K1}]+j_{\text{depS6K1}}} \quad \text{(S18)}$$

$$[\text{S6K1}] = [\text{S6K1}_\text{T}] - [\text{p-S6K1}] \tag{S19}$$

$$\frac{d[\text{mTORC2}_\text{T}]}{dt} = k_{\text{smTORC2T1}} + k_{\text{smTORC2T2}} \frac{{j_{\text{smTORC2T2}}}^4}{[\text{Sox2}]^4 + {j_{\text{smTORC2T2}}}^4} - d_{\text{mTORC2T}}[\text{mTORC2}_\text{T}] \tag{S20}$$

$$\frac{d[\text{p-mTORC2}]}{dt} = k_{\text{acmTORC2}} \frac{[\text{p-PI3K}]}{[\text{p-PI3K}] + j_{\text{pmTORC2}}} \frac{[\text{mTORC2}]}{[\text{mTORC2}] + j_{\text{acmTORC2}}} - k_{\text{depmTORC2}} \frac{[\text{p-mTORC2}]}{[\text{p-mTORC2}] + j_{\text{depmTORC2}}} \tag{S21}$$

$$[\text{mTORC2}] = [\text{mTORC2}_\text{T}] - [\text{p-mTORC2}] \tag{S22}$$

$$\frac{d[\text{GLUT1}]}{dt} = k_{\text{sGLUT10}} + k_{\text{sGLUT11}} \frac{[\text{HIF-1}\alpha]^4}{[\text{HIF-1}\alpha]^4 + {j_{\text{sGLUT11}}}^4} + k_{\text{sGLUT12}} \frac{[\text{OCT4}]^4}{[\text{OCT4}]^4 + {j_{\text{sGLUT12}}}^4} - d_{\text{GLUT1}}[\text{GLUT1}] \tag{S23}$$

$$\frac{d[\text{Glucose}_\text{in}]}{dt} = (k_{\text{siGlucose1}} + k_{\text{siGlucose2}}[\text{GLUT1}])[\text{Glucose}_\text{out}] - d_{\text{iGlucose}}[\text{Glucose}_\text{in}] \tag{S24}$$

$$\frac{d[\text{NADH}]}{dt} = k_{\text{sNADH1}} \frac{[\text{Glucose}_\text{in}]}{[\text{Glucose}_\text{in}] + j_{\text{sNADH1}}} [\text{NAD}^+] - k_{\text{dNADH2}} \frac{\text{ss}*\text{O}_2}{\text{ss}*\text{O}_2 + j_{\text{O2NADH}}} \frac{[\text{NADH}]}{[\text{NADH}] + j_{\text{dNADH2}}}$$
$$- k_{\text{dNADH1}} \frac{[\text{NADH}]}{[\text{NADH}] + j_{\text{dNADH1}}} \tag{S25}$$

$$[\text{NAD}^+] = [\text{NADH}_\text{T}] - [\text{NADH}] \tag{S26}$$

$$\frac{d[\text{CtBP}]}{dt} = k_{\text{sCtBP0}} + k_{\text{sCtBP1}} \frac{[\text{HIF-2}\alpha]^4}{[\text{HIF-2}\alpha]^4 + {j_{\text{sCtBP1}}}^4} + 2k_{\text{dCtBP2}}[\text{CtBP}_2] - d_{\text{CtBP}}[\text{CtBP}]$$
$$- k_{\text{sCtBP21}} \frac{[\text{NADH}]}{[\text{NADH}] + j_{\text{NADH}}} \frac{[\text{CtBP}]^2}{[\text{CtBP}]^2 + {j_{\text{sCtBP21}}}^2} \tag{S27}$$

$$\frac{d[\text{CtBP}_2]}{dt} = k_{\text{sCtBP21}} \frac{[\text{NADH}]}{[\text{NADH}] + j_{\text{NADH}}} \frac{[\text{CtBP}]^2}{[\text{CtBP}]^2 + {j_{\text{sCtBP21}}}^2} - d_{\text{CtBP2}}[\text{CtBP}_2] - k_{\text{dCtBP2}}[\text{CtBP}_2] \tag{S28}$$

$$\frac{d[\text{OCT4}]}{dt} = k_{\text{sOCT40}} + k_{\text{sOCT41}} \frac{[\text{HIF-2}\alpha]^4}{[\text{HIF-2}\alpha]^4 + {j_{\text{sOCT41}}}^4} \frac{[\text{CtBP}_2]^2}{[\text{CtBP}_2]^2 + {j_{\text{sOCT4}}}^2} + k_{\text{sOCT42}} \frac{[\text{HIF-2}\alpha]^4}{[\text{HIF-2}\alpha]^4 + {j_{\text{sOCT42}}}^4}$$
$$- k_{\text{deOCT4}}[\text{p-S6K1}] \frac{[\text{OCT4}]}{[\text{OCT4}] + j_{\text{deOCT4}}} - d_{\text{OCT4}}[\text{OCT4}] \tag{S29}$$

$$\frac{d[\text{Sox2}]}{dt} = k_{\text{sSox20}} + k_{\text{sSox21}} \frac{[\text{OCT4}]^4}{[\text{OCT4}]^4 + {j_{\text{sSox21}}}^4} - d_{\text{Sox2}}[\text{Sox2}] \tag{S30}$$

SUPPLEMENTAL TABLE S1: DESCRIPTION AND INITIAL VALUES OF VARIABLES

Variable	Description	Initial value (μM)
[HIF-1α]	Concentration of HIF-1α	1.10571
[PHD-2_T]	Total concentration of PHD-2 and PHD-2*	8.17486
[PHD-2*]	Concentration of active PHD-2*	5.67118
[HIF-2α]	Concentration of HIF-2α	1.25576
[PHD-3_T]	Total concentration of PHD-3 and PHD-3*	2.90121
[PHD-3*]	Concentration of active PHD-3*	1.17653
[EGF]	Concentration of EGF	0.02471
[p-PI3K]	Concentration of active p-PI3K	2.81856
[p-AKT]	Concentration of active p-AKT	2.16222
[REDD1]	Concentration of REDD1	0.05217
[mTORC1_T]	Total concentration of mTORC1 and p-mTORC1	10
[p-mTORC1]	Concentration of active p-mTORC1	9.03797
[p-S6K1]	Concentration of active S6K1	5.42844
[mTORC2_T]	Total concentration of mTORC2 and p-mTORC2	10
[p-mTORC2]	Concentration of active p-mTORC2	1.57358
[GLUT1]	Concentration of GLUT1	0.15413
[$Glucose_{in}$]	Concentration of intracellular Glucose	0.61559
[NADH]	Concentration of NADH	0.0255
[CtBP]	Concentration of CtBP	0.01224
[$CtBP_2$]	Concentration of CtBP dimer	3.1738E-7
[OCT4]	Concentration of OCT4	0.0013
[Sox2]	Concentration of Sox2	0.01

SUPPLMENTAL TABLE S2: PARAMETERS OF THE MODEL

Variable	Description	Value	Reference
O_2	Volume percentage of O_2 in air	[0%, 21%]	
ss	Value of O_2 level in μM corresponds to 1% O_2	9.86	[1]
j_{O2PHD2}	Threshold of O_2 for PHD-2 activation	250	[2]
j_{O2PHD3}	Threshold of O_2 for PHD-3 activation	400	Assumed
$k_{sHIF1\alpha}$	Production rate of HIF-1α mediated by p-S6K1	0.75 min^{-1}	Assumed
$k_{deHIF1\alpha}$	PHD-2*-dependent hydroxylation rate of HIF-1α	2 min^{-1}	Assumed
$j_{deHIF1\alpha}$	Michaelis constant for HIF-1α as a substrate of PHD-2*	2 μM	[3]
$d_{HIF1\alpha}$	Basal degradation rate of HIF-1α	0.03 min^{-1}	[4]
$k_{sPHD2T0}$	Basal production rate of PHD-2_T	0.00001 μM/min	Assumed
$k_{sPHD2T1}$	HIF-1α-dependent production rate of PHD-2_T	0.03 μM/min	[5]
$j_{sPHD2T1}$	Michaelis constant of HIF-1α-dependent PHD-2_T production	1 μM	Assumed
d_{PHD2T}	Basal degradation rate of PHD-2_T	0.0022 min^{-1}	Assumed
k_{acPHD2}	Activation rate of PHD-2	5 min^{-1}	Assumed
k_{dePHD2}	Inactivation rate of PHD-2*	1 min^{-1}	[3]
$k_{sHIF2\alpha}$	p-mTORC2-dependent production rate of HIF-2α	0.06 μM/min	Assumed
$j_{sHIF2\alpha}$	Michaelis constant of p-mTORC2-dependent HIF-2α production	2.1 μM	Assumed
$k_{deHIF2\alpha}$	PHD-3*-dependent hydroxylation rate of HIF-2α	0.1 min^{-1}	Assumed
$j_{deHIF2\alpha}$	Michaelis constant for HIF-2α as a substrate of PHD-3*	10 μM	[6]
d_{HIF2a}	Basal degradation rate of HIF-2α	0.001 min^{-1}	Assumed
$k_{sPHD3T0}$	Basal production rate of PHD-3_T	0.001 μM/min	Assumed
$k_{sPHD3T1}$	HIF-1α-dependent production rate of PHD-3_T	0.2 μM/min	Assumed
$j_{sPHD3T1}$	Michaelis constant of HIF-1α-dependent PHD-3_T production	1.5 μM	Assumed
$k_{sPHD3T2}$	HIF-2α-dependent production rate of PHD-3_T	0.085 μM/min	Assumed
$j_{sPHD3T2}$	Michaelis constant of HIF-2α-dependent PHD-3_T production	2 μM	Assumed
d_{PHD3T}	Basal degradation rate of PHD-3_T	0.02 min^{-1}	Assumed
k_{acPHD3}	Activation rate of PHD-3	2 min^{-1}	Assumed
k_{dePHD3}	Inactivation rate of PHD-3*	1 min^{-1}	Assumed
$k_{sREDD10}$	Basal production rate of REDD1	0.01 μM/min	Assumed
$k_{sREDD11}$	HIF-1α-dependent production rate of REDD1	0.5 μM/min	Assumed
$j_{sREDD11}$	Michaelis constant of HIF-1α-dependent REDD1 production	5 μM	Assumed
$k_{sREDD12}$	HIF-2α-dependent production rate of REDD1	0.5 μM/min	Assumed
$j_{sREDD12}$	Michaelis constant of HIF-2α-dependent REDD1 production	3 μM	Assumed
d_{REDD1}	Basal degradation rate of REDD1	0.5 min^{-1}	Assumed
k_{sEGF0}	Basal production rate of EGF	0.0001 μM/min	Assumed
k_{sEGF1}	HIF-1α-dependent production rate of EGF	1 μM/min	Assumed
j_{sEGF1}	Michaelis constant of HIF-1α-dependent EGF production	8 μM	Assumed
k_{sEGF2}	HIF-2α-dependent production rate of EGF	3 μM/min	Assumed
j_{sEGF2}	Michaelis constant of HIF-2α-dependent EGF production	5 μM	Assumed
d_{EGF}	Basal degradation rate of EGF	0.5 min^{-1}	Assumed
$PI3K_T$	Total concentration of PI3K and p-PI3K	10 μM	Assumed
k_{sPI3K}	Phosphorylation rate of PI3K	1 μM/min	Assumed
GF_0	Endogenous growth factor	1 μM	Assumed
j_{GF}	Michaelis constant of GF-dependent PI3K phosphorylation	9 μM	Assumed
j_{PI3K}	Michaelis constant of PI3K phosphorylation	1.5 μM	Assumed
d_{PI3K}	Dephosphorylation rate of p-PI3K	0.075 min^{-1}	Assumed
AKT_T	Total concentration of AKT and p-AKT	10 μM	Assumed
k_{acAKT}	p-PI3K-dependent phosphorylation rate of AKT	0.4 μM/min	Assumed
k_{acAKT1}	p-mTORC2-dependent phosphorylation rate of AKT	0.5 μM/min	Assumed
j_{pAKT2}	Michaelis constant of p-PI3K-dependent AKT phosphorylation	4 μM	Assumed

SUPPLEMENTAL TABLE S2-CONTINUED

Variable	Description	Value	Reference
j_{pAKT1}	Michaelis constant of p-mTORC2-dependent AKT phosphorylation	3 μM	Assumed
j_{acAKT}	Michaelis constant of AKT phosphorylation	10 μM	Assumed
k_{deAKT}	Dephosphorylation rate of p-AKT	0.2 μM/min	[7]
j_{deAKT}	Michaelis constant of p-AKT dephosphorylation	2 μM	[7]
$k_{smTORC1T1}$	Basal production rate of $mTORC1_T$	0.05 μM/min	Assumed
$k_{smTORC1T2}$	Sox2-dependent production rate of $mTORC1_T$	0.05 μM/min	Assumed
$j_{smTORC1T2}$	Michaelis constant of Sox2-dependent $mTORC1_T$ production	3 μM	Assumed
$d_{mTORC1T}$	Basal degradation rate of $mTORC1_T$	0.01 min^{-1}	Assumed
$k_{acmTORC1}$	Phosphorylation rate of mTORC1	3 μM/min	Assumed
$j_{pmTORC1}$	Michaelis constant of p-AKT-dependent mTORC1 phosphorylation	1 μM	Assumed
$j_{acmTORC1}$	Michaelis constant of mTORC1 phosphorylation	2 μM	Assumed
$k_{depmTORC11}$	Dephosphorylation rate of p-mTORC1	1 μM/min	Assumed
$j_{depmTORC11}$	Michaelis constant of p-mTORC1 dephosphorylation	5 μM	Assumed
$k_{depmTORC12}$	Dephosphorylation rate of REDD1-dependent p-mTORC1	1 min^{-1}	Assumed
$j_{depmTORC12}$	Michaelis constant of REDD1-dependent p-mTORC1 dephosphorylation	12 μM	Assumed
$S6K1_T$	Total concentration of S6K1 and p-S6K1	10 μM	Assumed
k_{acS6K1}	Phosphorylation rate of S6K1	0.2 min^{-1}	Assumed
j_{acS6K1}	Michaelis constant of S6K1 phosphorylation	15 μM	Assumed
$k_{depS6K1}$	Dephosphorylation rate of S6K1	0.5 μM/min	Assumed
$j_{depS6K1}$	Michaelis constant of S6K1 dephosphorylation	1 μM	Assumed
$k_{smTORC2T1}$	Basal production rate of $mTORC2_T$	0.09 μM/min	Assumed
$k_{smTORC2T2}$	Sox2-dependent production rate of $mTORC2_T$	0.01 μM/min	Assumed
$j_{smTORC2T2}$	Michaelis constant of Sox2-dependent $mTORC2_T$ production	8 μM	Assumed
$d_{mTORC2T}$	Basal degradation rate of $mTORC2_T$	0.01 min^{-1}	Assumed
$k_{acmTORC2}$	Phosphorylation rate of mTORC2	2 μM/min	Assumed
$j_{pmTORC2}$	Michaelis constant of p-PI3K-dependent mTORC2 phosphorylation	8.2 μM	Assumed
$j_{acmTORC2}$	Michaelis constant of mTORC2 phosphorylation	9.2 μM	Assumed
$k_{depmTORC2}$	Dephosphorylation rate of p-mTORC2	0.4 μM/min	Assumed
$j_{depmTORC2}$	Michaelis constant of p-mTORC2 dephosphorylation	1 μM	Assumed
$k_{sGLUT10}$	Basal production rate of GLUT1	0.01 μM/min	Assumed
$k_{sGLUT11}$	HIF-1α-dependent production rate of GLUT1	2 μM/min	Assumed
$j_{sGLUT11}$	Michaelis constant of HIF-1α-dependent production of GLUT1	3 μM	[8]
$k_{sGLUT12}$	OCT4-dependent production rate of GLUT1	6 μM/min	Assumed
$j_{sGLUT12}$	Michaelis constant of OCT4-dependent production of GLUT1	1 μM	Assumed
d_{GLUT1}	Degradation rate of GLUT1	0.3 min^{-1}	Assumed
$Glucose_{out}$	Concentration of extracellular Glucose	10 μM	[8]
$k_{siGlucose1}$	Transportation rate of Glucose	0.02 min^{-1}	Assumed
$k_{siGlucose2}$	GLUT1-dependent transportation rate of Glucose	0.03 $\mu M^{-1} min^{-1}$	Assumed
$d_{iGlucose}$	Degradation rate of $Glucose_{in}$	0.4 min^{-1}	Assumed
$NADH_T$	Total concentration of NADH and NAD^+	10 μM	Assumed
k_{sNADH1}	Production rate of NADH	0.08 min^{-1}	Assumed
j_{sNADH1}	Michaelis constant of NADH production	10 μM	Assumed
k_{dNADH1}	Degradation rate of NADH via glycolysis	0.2 μM/min	Assumed
j_{dNADH1}	Michaelis constant of NADH degradation via glycolysis	7 μM	Assumed
j_{O2NADH}	Threshold of O_2 for NADH production via oxidative phosphorylation	50	Assumed

SUPPLEMENTAL TABLE S2-CONTINUED

Variable	Description	Value	Reference
k_{dNADH2}	Degradation rate of NADH via oxidative phosphorylation	0.5 μM/min	Assumed
j_{dNADH2}	Michaelis constant of NADH degradation via oxidative phosphorylation	0.2 μM	Assumed
k_{sCtBP0}	Basal production rate of CtBP	0.001 μM/min	Assumed
k_{sCtBP1}	HIF-2α-dependent production rate of CtBP	0.9 μM/min	Assumed
j_{sCtBP1}	Michaelis constant of HIF-2α-dependent CtBP production	10 μM	Assumed
d_{CtBP}	Degradation rate of CtBP	0.1 min^{-1}	Assumed
$k_{sCtBP21}$	Production rate of $CtBP_2$	8 min^{-1}	Assumed
j_{NADH}	Michaelis constant of NADH-dependent $CtBP_2$ production	15 μM	Assumed
$j_{sCtBP21}$	Michaelis constant of CtBP-dependent $CtBP_2$ production	8 μM	Assumed
d_{CtBP2}	Degradation rate of $CtBP_2$	0.1 min^{-1}	Assumed
k_{dCtBP2}	Conversion rate of $CtBP_2$ to CtBP	0.0001 min^{-1}	Assumed
k_{sOCT40}	Basal production rate of OCT4	0.003 μM/min	Assumed
k_{sOCT41}	HIF-2α-$CtBP_2$-dependent production rate of OCT4	1 μM/min	Assumed
j_{sOCT41}	Michaelis constant of HIF-2α-$CtBP_2$ dependent $CtBP_2$ production	8 μM	Assumed
j_{sOCT4}	Michaelis constant of $CtBP_2$-dependent $CtBP_2$ production	0.1 μM	Assumed
k_{sOCT42}	HIF-2α-dependent production rate of OCT4	0.3 μM/min	Assumed
j_{sOCT42}	Michaelis constant of HIF-2α-dependent $CtBP_2$ production	10 μM	Assumed
k_{deOCT4}	p-S6K1-dependent degradation rate of OCT4	0.2 min^{-1}	Assumed
j_{deOCT4}	Michaelis constant of p-S6K1-dependent OCT4 degradation	6 μM	Assumed
d_{OCT4}	Degradation rate of OCT4	0.1 min^{-1}	Assumed
k_{sSox20}	Basal production rate of Sox2	0.001 μM/min	Assumed
k_{sSox21}	OCT4-dependent production rate of Sox2	0.8 μM/min	Assumed
j_{sSox21}	Michaelis constant of OCT4-dependent Sox2 production	9 μM	Assumed
d_{Sox2}	Degradation rate of Sox2	0.1 min^{-1}	Assumed

REFERENCES

1. Qutub, A.A.; Popel, A.S. A computational model of intracellular oxygen sensing by hypoxia-inducible factor HIF1 alpha. *J Cell Sci* **2006**, *119*, 3467-3480, doi:10.1242/jcs.03087.
2. Koivunen, P.; Hirsilä, M.; Günzler, V.; Kivirikko, K.I.; Myllyharju, J. Catalytic properties of the asparaginyl hydroxylase (FIH) in the oxygen sensing pathway are distinct from those of its prolyl 4-hydroxylases. *J Biol Chem* **2004**, *279*, 9899-9904, doi:10.1074/jbc.M312254200.
3. Wang, P.; Guan, D.; Zhang, X.-P.; Liu, F.; Wang, W. Modeling the regulation of p53 activation by HIF-1 upon hypoxia. *FEBS Letters* **2019**, *593*, 2596-2611, doi:10.1002/1873-3468.13525.
4. Daleprane, J.B.; Daleprane, J.B.; Schmid, T.; Dehne, N.; Rudnicki, M.; Menrad, H.; Geis, T.; Ikegaki, M.; Ong, T.P.; Brüne, B.; et al. Suppression of hypoxia-inducible factor-1α contributes to the antiangiogenic activity of red propolis polyphenols in human endothelial cells. *J Nutr* **2012**, *142*, 441-447, doi:10.3945/jn.111.150706.
5. Bagnall, J.; Leedale, J.; Taylor, S.E.; Spiller, D.G.; White, M.R.; Sharkey, K.J.; Bearon, R.N.; Sée, V. Tight control of hypoxia-inducible factor-α transient dynamics is essential for cell survival in hypoxia. *J Biol Chem* **2014**, *289*, 5549-5564, doi:10.1074/jbc.M113.500405.
6. Hirsilä, M.; Koivunen, P.; Günzler, V.; Kivirikko, K.I.; Myllyharju, J. Characterization of the Human Prolyl 4-Hydroxylases That Modify the Hypoxia-inducible Factor. *Journal of Biological Chemistry* **2003**, *278*, 30772-30780, doi:10.1074/jbc.m304982200.
7. Wee, K.B.; Surana, U.; Aguda, B.D. Oscillations of the p53-Akt network: implications on cell survival and death. *PLoS One* **2009**, *4*, e4407, doi:10.1371/journal.pone.0004407.
8. Wang, P.; Zhang, X.-P.; Liu, F.; Wang, W. Progressive Deactivation of Hydroxylases Controls Hypoxia-Inducible Factor-1α-Coordinated Cellular Adaptation to Graded Hypoxia. *Research* **2025**, *8*, doi:10.34133/research.0651.

Supplemental Figures:

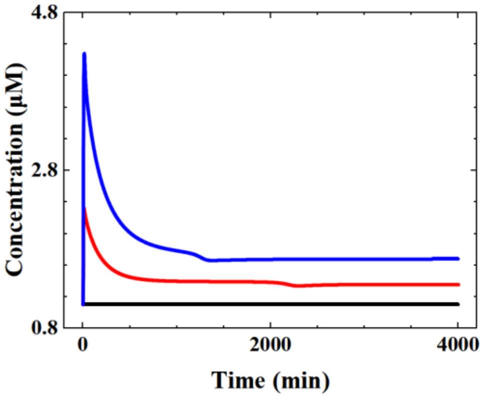

Figure S1. Dynamics of HIF-1α at different oxygen levels. Time-courses of [HIF-1α] under L_{O2} = 21% (black), 5% (red), and 3% (blue).

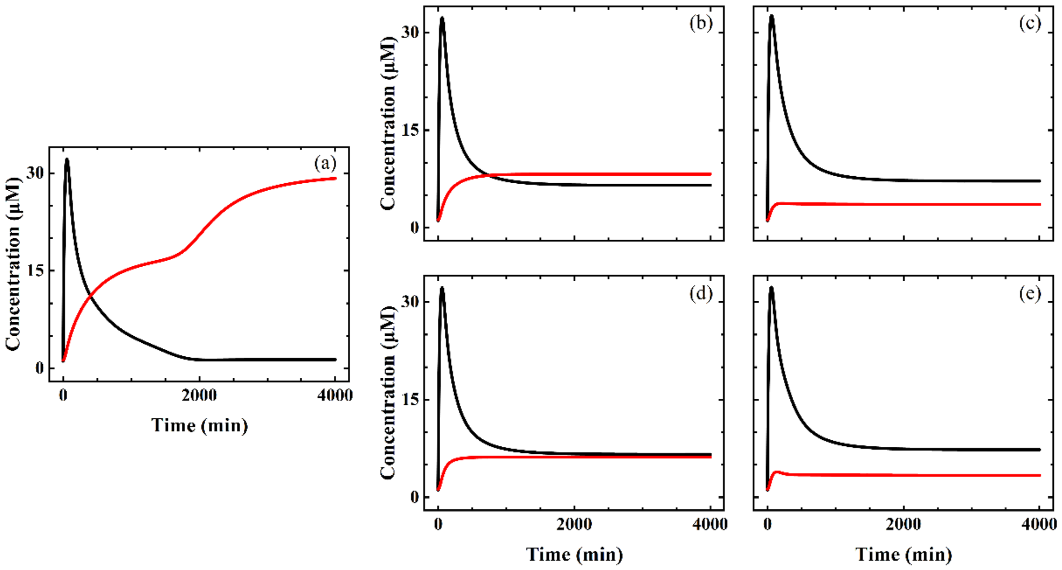

Figure S2. Effect of PHD3 on the HIF switch. Time-coursesof [HIF-1α] (black) and [HIF-2α] (red) at L_{O2} = 1% under the following parameter settings: (a) $k_{sPHD3T1}$ = 0.2 and $k_{sPHD3T2}$ = 0.085 (control); (b) $k_{sPHD3T1}$ = 0.4 and $k_{sPHD3T2}$ = 0.085; (c) $k_{sPHD3T1}$ = 0.8 and $k_{sPHD3T2}$ = 0.085; (d) $k_{sPHD3T1}$ = 0.2 and $k_{sPHD3T2}$ = 0.4; and (e) $k_{sPHD3T1}$ = 0.2 and $k_{sPHD3T2}$ = 0.8.

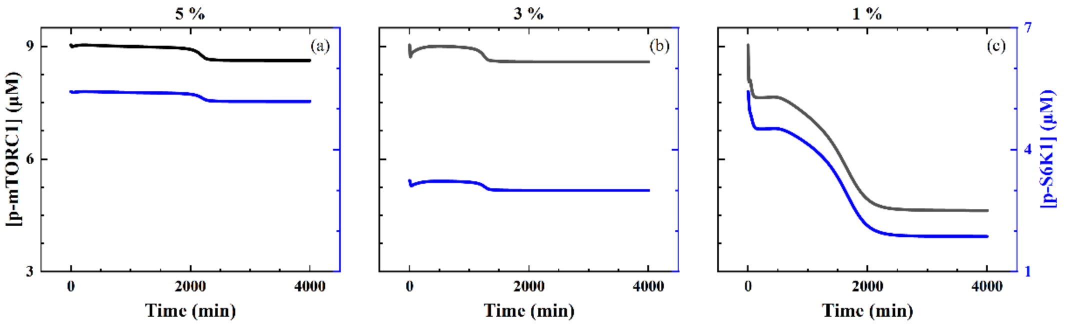

Figure S3. Dynamics of the key nodes within the positive OCT4–SOX2–mTORC1–S6K1 feedback loop. Time-courses of [p-mTORC1] (black) and [p-S6K1] (blue) for L_{O2} = 5% (a), 3% (b), and 1% (c).

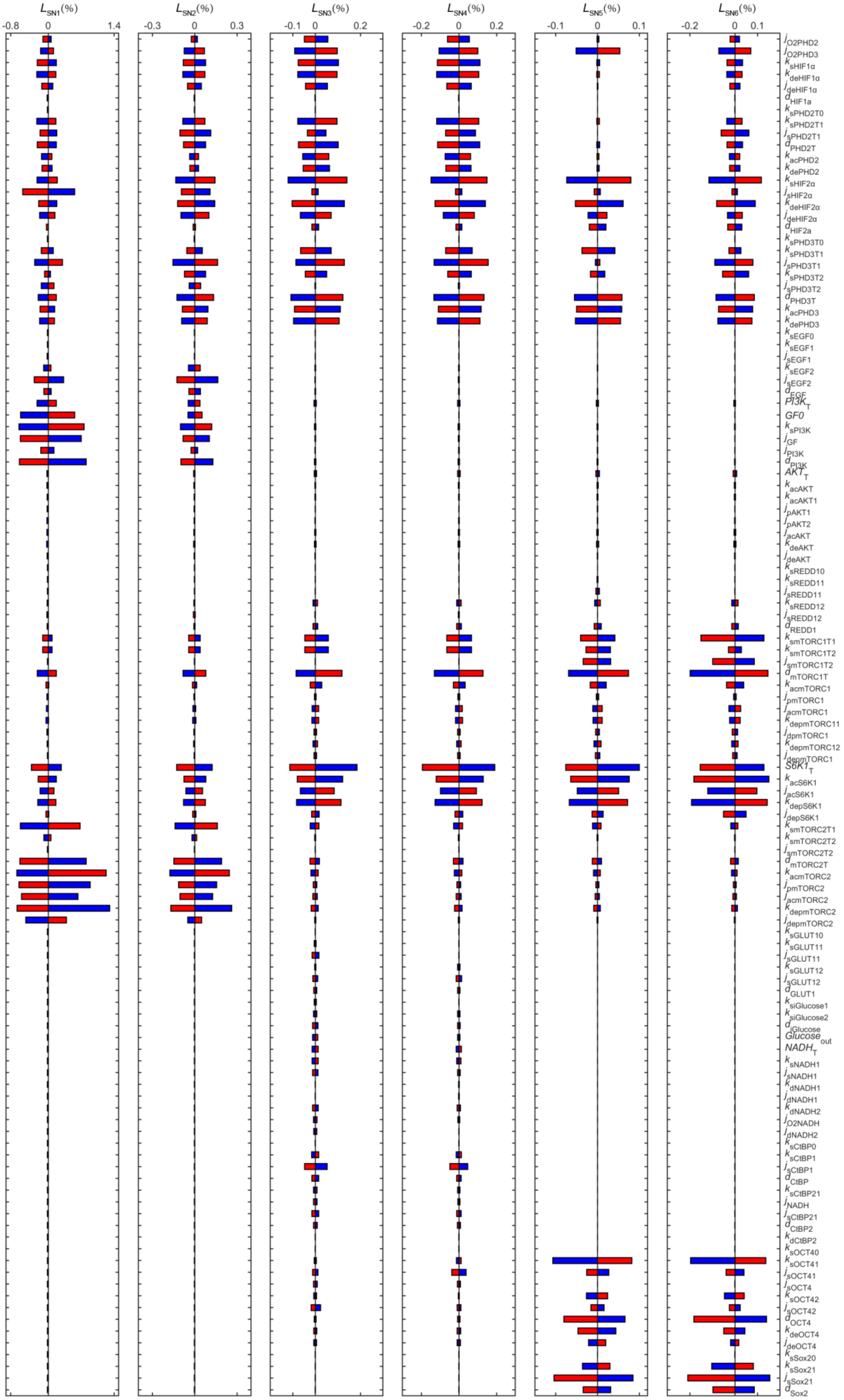

L_SN1(%)
-0.8
0
1.4
L_SN2(%)
-0.3
0
0.3
L_SN3(%)
-0.1
0
0.2
L_SN4(%)
-0.2
0
0.2
L_SN5(%)
-0.1
0
0.1
L_SN6(%)
-0.2
0
0.1
j_O2PHD2
j_O2PHD3
k_sHIF1α
k_deHIF1α
j_deHIF1α
d_HIF1a
k_sPHD2T0
k_sPHD2T1
j_sPHD2T1
d_PHD2T
k_acPHD2
k_dePHD2
k_sHIF2α
j_sHIF2α
k_deHIF2α
j_deHIF2α
d_HIF2a
k_sPHD3T0
k_sPHD3T1
j_sPHD3T1
k_sPHD3T2
j_sPHD3T2
d_PHD3T
k_acPHD3
k_dePHD3
k_sEGF0
k_sEGF1
j_sEGF1
k_sEGF2
j_sEGF2
d_EGF
PI3K_T
GF0
k_sPI3K
j_GF
j_PI3K
d_PI3K
AKT_T
k_acAKT
k_acAKT1
j_pAKT1
j_pAKT2
j_acAKT
k_deAKT
j_deAKT
k_sREDD10
k_sREDD11
j_sREDD11
k_sREDD12
j_sREDD12
d_REDD1
k_smTORC1T1
k_smTORC1T2
j_smTORC1T2
d_mTORC1T
k_acmTORC1
j_pmTORC1
j_acmTORC1
k_depmTORC11
j_dpmTORC1
k_depmTORC12
j_depmTORC1
S6K1_T
k_acS6K1
j_acS6K1
k_depS6K1
j_depS6K1
k_smTORC2T1
k_smTORC2T2
j_smTORC2T2
d_mTORC2T
k_acmTORC2
j_pmTORC2
j_acmTORC2
k_depmTORC2
j_depmTORC2
k_sGLUT10
k_sGLUT11
j_sGLUT11
k_sGLUT12
j_sGLUT12
d_GLUT1
k_siGlucose1
k_siGlucose2
d_Glucose
Glucose_out
NADH_T
k_sNADH1
j_sNADH1
k_dNADH1
j_dNADH1
k_dNADH2
j_O2NADH
j_dNADH2
k_sCtBP0
k_sCtBP1
j_sCtBP1
d_CtBP
k_sCtBP21
j_NADH
j_sCtBP21
d_CtBP2
k_dCtBP2
k_sOCT40
k_sOCT41
j_sOCT41
j_sOCT4
k_sOCT42
j_sOCT42
d_OCT4
k_deOCT4
j_deOCT4
k_sSox20
k_sSox21
j_sSox21
d_Sox2

Figure S4. Assessment of the system's robustness to the emergence of the four stable states. The sensitivity of the oxygen threshold (L_{SN}) for each SN (including SN_1-SN_6) to ±5% variations (compared to the default setting) in each of the 116 parameters is assessed.
